# Evaluation of Psychological Stress Parameters in Coronary Patients by Three Different Questionnaires as Pre-Requisite for Comprehensive Rehabilitation

**DOI:** 10.3390/brainsci10050316

**Published:** 2020-05-22

**Authors:** Ana Maria Pah, Nicoleta Florina Buleu, Anca Tudor, Ruxandra Christodorescu, Dana Velimirovici, Stela Iurciuc, Maria Rada, Gheorghe Stoichescu-Hogea, Marius Badalica-Petrescu, Doina Georgescu, Dorina Nutiu, Mircea Iurciuc, Simona Dragan

**Affiliations:** 1 Department of Cardiology, “Victor Babes” University of Medicine and Pharmacy, 300041 Timisoara, Romania; ana11p@yahoo.com (A.M.P.); buleu.florina@gmail.com (N.F.B.); danavelimirovici@yahoo.com (D.V.); stela_iurciuc@yahoo.com (S.I.); radamariam@gmail.com (M.R.); goguhogea@yahoo.com (G.S.-H.); marius_badalica@yahoo.com (M.B.-P.); mirceaiurciuc@gmail.com (M.I.); simona.dragan@umft.ro (S.D.); 2 Institute of Cardiovascular Diseases, 300020 Timisoara, Romania; florina_28nick@yahoo.com; 3 Department of Functional Sciences, “Victor Babes” University of Medicine and Pharmacy, 300041 Timisoara, Romania; 4 Department of Internal Medicine, “Victor Babes” University of Medicine and Pharmacy, 300041 Timisoara, Romania; doinageox@gmail.com

**Keywords:** coronary artery disease, anxiety, depression, psychological stress parameters, comprehensive rehabilitation

## Abstract

Background: Negative psychological conditions are common in patients with cardiovascular diseases. Although depression has been scrutinized over the years in these patients, only recently has anxiety emerged as another important risk factor. The purpose of this study was to compare the parameters of psychological stress in a population of coronary patients with and without myocardial revascularization procedures and to analyze lifestyle and socio-economic contributors to the state of health of these patients before inclusion in a comprehensive individualized rehabilitation program. Methods: This study included 500 patients with coronary artery disease (CAD) in stable condition divided in 2 groups: 200 patients who underwent coronary artery bypass grafting (CABG) or percutaneous transluminal coronary angioplasty (PTCA) (Group 1) and 300 patients without myocardial revascularization (Group 2) with stable angina or thrombolyzed myocardial infarction. The protocol included screening for anxiety/depression after procedure using three different scales: Duke Anxiety-Depression Scale, Hospital Anxiety and Depression Scale (HADS) and the Type D Personality Scale (DS-14) scale that evaluates negative affectivity (NA) and social inhibition (SI). Results: Significant differences between groups were observed for HAD-A (9.1 ± 4.18 for Group 1 vs. 7.8 ± 4.03 for Group 2, *p* = 0.002) and DUKE scores (30.2 ± 12.25 for Group 1 vs. 22.7 ± 12.13 for Group 2, *p* < 0.001). HAD-A scores (*p* = 0.01) and DUKE scores (*p* = 0.04) were significantly higher in patients who underwent PTCA vs. CABG. CAD patients without myocardial revascularization (Group 2, *n* = 300) presented anxiety in proportion of 72.3% (*n* = 217) out of which 10.7% (*n* = 32) had severe anxiety, and 180 patients had depression (a proportion of 60%) out of which 1.3% (*n* = 4) presented severe depression. The correlation between the presence of type 2 diabetes mellitus (T2DM) and type D personality in revascularized patients (*n* = 200) was significant (Chi2 test, *p* = 0.010). By applying multinomial regression according to the Cox and Snell R-square model and multivariate linear regression by the Enter method, we demonstrated that male gender, age and marital status proved significant predictors for psychological stress in our study population. Conclusions: The results obtained in this study provide a framework for monitoring anxiety, depression and type D personality in coronary patients before inclusion in comprehensive rehabilitation programs. Behavioral and psychological stress responses in patients with CAD significantly correlate with risk factors, and could influence the evolution of the disease. Moreover, other factors like gender, income and marital status also seem to play a decisive role. Evaluation of psychological stress parameters contributes to a better individualization at the start of these programs, because it allows adjusting of all potential factors that may influence positive outcomes.

## 1. Introduction

Since 2010, cardiovascular diseases (CVD) are the leading cause of death worldwide, resulting in over 7 million deaths. They can affect individuals of any age but become more common in older ages, tripling with each decade of life. More than half of all deaths caused by CVD occur in patients aged over 70 years [1].

The increased incidence of CVD is due to the consumption of foods with high caloric content, sedentary lifestyle, alcohol intake, smoking and psychosocial stress, which all cause economic and social problems for the patient himself and the patient’s family and employer, often limiting work capacity [2].

Recently, numerous epidemiological studies have proven that psychosocial factors, especially anxiety and depression, are associated with an increased risk of developing coronary artery disease (CAD) [3,4]. In these studies, various assessment scales were used to measure psychological stress [5]. At childbearing age, women have the highest prevalence of psychiatric disorders [6,7]. Furthermore, anxiety and depression severity has a tendency to be higher in women with myocardial infarction [8]. 

The Hospital Anxiety and Depression Scale (HADS) is a self-assessment tool commonly used in non-psychiatric patients to assess psychological stress. It was developed by Zsigmond and Snaith more than 30 years ago [9]. Since then, numerous studies have recommended HADS to assess psychological distress among CVD patients [10,11] or in the general population [12,13]. 

The Duke Anxiety-Depression Scale (DUKE) has seven items that measure symptoms of depression and anxiety, and seems to be a reliable instrument for detecting psychological stress [14]. Type D personality combines two stable traits measured by the DS-14 questionnaire, the tendency to experience negative emotions (negative affectivity, NA) together with the tendency not to share these emotions in social interactions, because of fear of rejection or disapproval (social inhibition, SI) [15]. Type D personality is a vulnerability factor for future psychological stress, which has been associated with anxiety and depression in CAD patients, even at 10 years of follow-up after myocardial revascularization procedures [16]. Burkauskas et al. observed, in a study performed on 510 patients with CAD included in a cardiac rehabilitation program, that depression, anxiety and type D personality were associated with worsening cognitive performance, independent of the severity of CAD and socio-demographic characteristics [5].

The purpose of this study was to compare parameters of psychological stress in CAD patients with and without myocardial revascularization procedures and to analyze all lifestyle contributors that might influence this type of stress, including socio-economic status. To the best of our knowledge, the present study is the first that aims to investigate the incidence of anxiety, depression and type D personality by three different questionnaires, with the intention to better individualize coronary patients for cardiac rehabilitation.

## 2. Material and Methods

### 2.1. Study Design and Patient Population

This retrospective study was conducted between 2018 and 2019 in the Cardiovascular Prevention and Rehabilitation Clinic of the Institute of Cardiovascular Diseases Timisoara, Romania, on 500 consecutive hospitalized coronary patients who underwent diagnostic coronary angiography, followed by revascularization procedures in selected cases, as documented by individual case histories. Coronary artery disease was classified as monovascular, bivascular or trivascular, and revascularization procedures were coronary artery bypass grafting (CABG) or percutaneous transluminal coronary angioplasty (PTCA). All patients were divided into two groups, depending on the presence or absence of myocardial revascularization procedures. Group 1 (*n* = 200) included CAD patients with myocardial revascularization procedures and Group 2 (*n* = 300) included those without myocardial revascularization procedures and with stable angina or thrombolyzed myocardial infarction. In Group 1, 119 patients underwent CABG and 81 patients PTCA. All patients voluntarily participated in the psychological evaluation. 

Patients with secondary hypertension, chronic kidney disease, inflammatory diseases, known infections or neoplasms, mental illness or history of prophylactics for mental illness were excluded from study. All patients maintained the previously prescribed cardiac medications at the same doses. The study was conducted with the approval of the local Ethics Commission. Informed consent was obtained in all cases.

### 2.2. Clinical and Biochemical Evaluation

Personal data including age, gender, marital status, income, medical history of premature CVD in first-degree relatives (age < 55 years for M subjects, age < 65 years for F subjects) and smoking status were taken. The clinical evaluation included measurement of systolic (SBP) and diastolic blood pressures (DBP) and body mass index (BMI). Blood pressure (BP) was determined according to the European Guidelines on cardiovascular disease prevention in clinical practice [17]. Body weight (W(kg)) was determined using a mechanical scale. Height (H(m)) was determined using a metal talimeter (Fazzini, Vimodrone, Italy). The body mass index (BMI (kg/m^2^)) was calculated according to the following formula: BMI = weight (kg) ÷ height^2^ (m^2^). Abdominal circumference (AC (cm)) was determined by a metallic centimeter. Abdominal obesity was defined as abdominal circumference > 94 cm in men and >80 cm in women [18]. For the determination of total cholesterol, triglycerides, LDL and HDL-cholesterol, photometric methods (Dimension RXL-MAX, Dade Behring, Erlangen, Germany) were used. For fasting blood glucose the enzymatic method with hexokinase (HK) was applied, using Siemens reagents on a Dimension RXL-MAX, Dade Behring device, Erlangen, Germany. For determination of renal parameters, a colorimetric method was used for uric acid and the Jaffe method without deproteinization for creatinine. Hyperuricemia was defined as serum uric acid (UA) ≥ 7 mg/dL in men and ≥ 6 mg/dL in women according to the European League Against Rheumatism Guide (EULAR) [19]. Estimated glomerular filtration rate (eGFR) was calculated based on MDRD (Modification of Diet in Renal Disease) formula [20]: eGFR = 186 × (Creatinine/88.4)^−1.154^ × (Age)^−0.203^ × (0.742 if female). Patients with type 2 diabetes mellitus (T2DM) were previously diagnosed according to the consensus report by the American Diabetes Association (ADA) and the European Association for the Study of Diabetes (EASD) based on fasting plasma glucose >126 mg/dL or 2 h plasma glucose >200 mg/dL by oral glucose tolerance test (OGTT) [21].

#### 2.2.1. Cardiac Evaluation

Transthoracic echocardiography was performed in all patients included in the study on a General Electric Vivid 9 ultrasound system, manufactured by GEMS Ultrasound, Tirat Carmel, Israel. Electrocardiographic recordings and chest X-rays were taken to all patients to certify their stable clinical condition at enrolment. Two-dimensional ultrasound provided morphological and functional information about: dimensions, cavities, heart walls, presence of valvular lesions, ejection fraction and systolic performance. Doppler ultrasound by the two variants, spectral (for quantitative applications) and color (for qualitative applications), appreciated and quantified valvular regurgitation, systolic and diastolic function of the left ventricle and pulmonary circulation pressures. Thus, cardiac ultrasound allowed the assessment of the morphology and function of the heart valves and the presence or absence of ischemic secondary cardiomyopathy. Furthermore, some kinetic disorders could be detected correlated with the presence of coronary heart disease.

#### 2.2.2. Assessment of Psychological Stress

Assessment of psychological stress was quantified using 3 different scales: Duke Anxiety-Depression Scale (DUKE), Hospital Anxiety and Depression Scale (HADS) and Type D Personality Scale (DS-14). The HADS [15] is composed of 14 items and contains two subscales, one for anxiety and another one for depression. Each item is quantified using a 4-point Likert scale, from 0 (no symptoms) to 3 (maximum symptom level). The maximum score for each subscale is 21, scores 0–7 on each subscale are considered normal, while scores above 11 signify a considerable psychological morbidity, either anxiety (HAD-A) or depression (HAD-D). Scores 8–10 indicate a borderline status. Scores were considered if at least 5 responses were given for each subscale. Missing answers in patients who completed only 5 or 6 items were replaced based on the sum of the filled items, multiplied by 7/5 or 7/6, respectively. The DS-14 scale [15] has 14 items, each evaluated on a scale between 0 = false and 4 = true and also using two subscales, one for social inhibition (SI) and one for negative affectivity (NA). The total score on each subscale is between 0 and 28. The use of the DS-14 scale is dichotomic, requiring a score ≥ 10 in both subclasses to fulfill the D personality condition. The DUKE scale [14] has eleven items that measure psychological symptoms (depressed feelings, nervousness), somatic symptoms (fatigue, sleep problems), self-esteem (comfortable around people, give up too easily) and cognition (difficulties in concentration). For correct scoring, the total score is multiplied by 7.143 to obtain the DUKE-AD score on a scale of 0 for lowest to 100 for highest symptom level. A score close to the value of 100 represents as good a state of health as possible.

### 2.3. Statistical Analysis

Statistical analysis was performed with EpiInfo software (v.7.2.2.6, CDC, Atlanta, Georgia, USA) and with SPSS software, (version 17, SPSS Inc., Chicago, USA). The data were electronically filed using Microsoft Excel (version 2013, MS Corp., Redmond, Washington, USA). For numeric variables descriptive statistics were performed and the comparisons between these were made with the non-parametric Kruskal–Wallis test or by determining the Spearman’s correlation coefficient for more than 2 series. The Mann–Whitney test was used for comparisons between two sets of values with no Gaussian distribution. For nominal variables, frequency tables were elaborated and the associations between these were achieved by applying the chi2 (χ2) test. The results were considered significant for a value of *p* < 0.05. Multinomial regression according to the Cox and Snell R-square model and multivariate linear regression (Enter method) were used to identify all potential predictors for psychological stress.

## 3. Results

In Table 1 the clinical, biochemical and demographic features of all CAD patients (*n* = 500) are summarized.

Significant differences were observed between CAD patients with myocardial revascularization (Group 1, *n* = 200) and CAD patients without myocardial revascularization (Group 2, *n* = 300) regarding age (62.3 ± 7.63 years for Group 1 vs. 64.2 ± 7.92 years for Group 2, *p* = 0.004), HAD-A (9.1 ± 4.18 for Group 1 vs. 7.8 ± 4.03 for Group 2, *p* = 0.002), DUKE score (30.2 ± 12.25 for Group 1 vs. 22.7 ± 12.13 for Group 2, *p* < 0.001), total cholesterol (167.5 ± 40.36 mg/dL for Group 1 vs. 195.7 ± 49.49 mg/dL for Group 2, *p* < 0.001), HDL-c (43.5 ± 13.29 mg/dL for Group 1 vs. 51.7 ± 13.17 mg/dL for Group 2, *p* < 0.001), LDL-c (93 ± 27.77 mg/dL for Group 1 vs. 112.6 ± 37.51 for Group 2, *p* < 0.001), triglycerides (136 ± 71.08 mg/dL for Group 1 vs. 158.3 ± 99.32 for Group 2, *p* = 0.001), systolic blood pressure (136.3 ± 22.14 mmHg for Group 1 vs. 148.6 ± 23.63 mmHg for Group 2, *p* < 0.001), diastolic blood pressure (77.7 ± 11.97 mmHg for Group 1 vs. 86.1 ± 13.06 mmHg for Group 2, *p* < 0.001), uric acid (5.3 ± 1.39 mg/dL for Group 1 vs. 5.8 ± 1.23 mg/dL for Group 2, *p* < 0.001), creatinine (1.1 ± 0.3 mg/dL for Group 1 vs. 1.3 ± 0.26 for Group 2, *p* < 0.001) and estimated glomerular filtration rate (58.3 ± 13.99 mL/min/1.73 m^2^ for Group 1 vs. 55.8 ± 15.17 mL/min/1.73 m^2^ Group 2, *p* = 0.019) (Table 1). Type D personality was observed in 63 patients (31.5%) in Group 1 and in 84 patients (28%) in Group 2. 

Regarding other cardiovascular risk factors, significant differences between groups were for male gender (*p* < 0.001), smoking (*p* < 0.001), obesity (*p* = 0.001), previous anxiolytic and antidepressant treatment (*p* = 0.039) and family medical history of premature CVD (*p* = 0.043) (Table 2).

There were significant differences in Group 1 (the revascularized group) regarding male gender (78.2% in CABG subgroup vs. 61.7% in PTCA subgroup, *p* = 0.012) and HAD-A score (7.1 ± 3.77 in CABG subgroup vs. 8.8 ± 4.2 in PTCA subgroup, *p* = 0.010). 

The HAD-A scores were significantly higher in patients who underwent PTCA vs. CABG (*p* = 0.01). DUKE scores were also significantly higher in patients who underwent PTCA vs. CABG (*p* = 0.04) (Table 3). 

In Group 1, severe anxiety was present in proportion of 13.6% (*n* = 12) and was significantly higher in patients with PTCA vs. CABG (Chi2 test, *p* = 0.027) (Table 4).

Results after applying HADS showed that CAD patients without myocardial revascularization (Group 2, *n* = 300) presented anxiety in a proportion of 72.3% (*n* = 217) out of which 10.7% (*n* = 32) had severe anxiety (Table 5).

By applying the Mann-Whitney U Test and Chi2 Test to patients with severe anxiety from both groups we observed that those without myocardial revascularization and without income (*n* = 6), had HAD-A score significantly higher compared to those with income (*n* = 26) (Table 6).

Moreover, we observed that in coronary patients with myocardial revascularization and without a partner (*n* = 5), HAD-A (*p* = 0.041) and DUKE (*p* = 0.037) scores were significantly different than in those with a partner (*n* = 7). Significant differences were also observed in coronary patients without myocardial revascularization with a partner (*n* = 18) and without a partner (*n* = 14) in terms of HAD-A (*p* = 0.049) and DUKE (*p* = 0.042) (Table 7).

**Figure 1:**
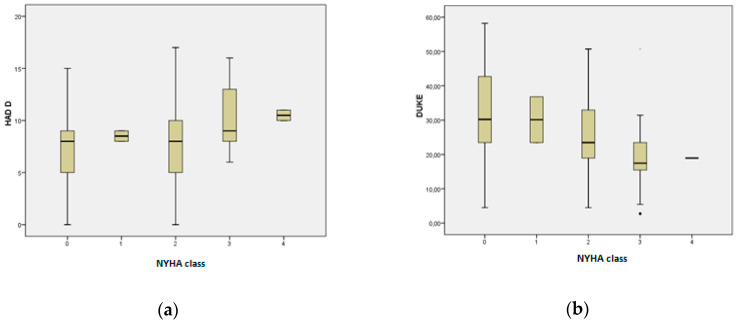
(**a**) Boxplots representing HAD-D values, compared to the NYHA functional class in Group 2 (*n* = 300); (*p <* 0.001). (**b**) Boxplots representing DUKE score values, compared to the NYHA functional class in Group 2 (*n* = 300); (*p* = 0.029).

By applying the Kruskal–Wallis nonparametric test it was observed that in Group 2 (*n* = 300) HAD-D values were significantly increased according to severity of NYHA (New York Heart Association) functional class (*p* = 0.029) (Figure 1a). DUKE score values decreased significantly with the increase of NYHA class (*p* < 0.001) (Figure 1b).

In CAD patients without myocardial revascularization (Group 2, *n* = 300), 180 patients had depression (a proportion of 60%) out of which 1.3% (*n* = 4) presented severe depression (Table 8). Risk analysis for the whole of the coronary patients in the study (*n* = 500), revealed that patients without a partner or with low income were at risk for developing severe depression (Chi2 test, *p* = 0.002, OR = 12.35, 95%CI = (2.36, 64.74)) and (Chi2 test, *p* = 0.008, OR = 4.10, 95%CI = (1.53, 10.93)), respectively (Table 9 and Table 10). 

Significant correlations were observed as follows (Table 11): increased values of HAD-A were significantly correlated, directly and weakly, with increased values of HbA1c (ρ = 0.23 and *p* = 0.001); increased HAD-D values were significantly correlated, directly and weakly, with blood glucose values (ρ = 0.151 and *p* = 0.033); increased values of the DUKE score correlated significantly, inversely and weakly with NYHA class (ρ = −0.143 and *p* = 0.044), directly and weakly with increased TC (ρ = 0.145 and *p* = 0.04) and HDL-c (ρ = 0.25 and *p* < 0.001); high values of DS-14 NA correlated significantly, directly and weakly with increased values of HbA1c (ρ = 0.186 and *p* = 0.008). 

Increased values of HAD-A were significantly correlated, inversely and weakly, with values of eGFR (ρ = −0.143 and *p* = 0.043); increased HAD-D values were significantly correlated, inversely and weakly, with blood glucose values (ρ = −0.160 and *p* = 0.024). Increased values of the DUKE score were significantly, inversely and weakly correlated with HT grades (ρ = −0.140 and *p* = 0.048); directly and weakly with increased values of DBP (ρ = 0.156 and *p* = 0.027), LVEF (ρ = 0.279 and *p* < 0.001) and with decreased values of eGFR (ρ = 0.272 and *p* < 0.001) (Table 12).

In revascularized patients with type 2 diabetes mellitus (*n* = 62), significantly increased HAD-A scores compared with scores of patients without diabetes mellitus were observed (Mann-Whitney test, *p* = 0.002). We also observed significantly increased DS-14 NA scores in revascularized patients with T2DM (Mann-Whitney test, *p* = 0.024) (Table 13). 

The correlation between the presence of T2DM and type D personality in revascularized patients (*n* = 200) was significant (Chi2 test, *p* = 0.010).

Finally, we applied multinomial regression according to the Cox and Snell R-square model including all patients (*n* = 500) using the HAD-A and HAD-D scores as dependent variables, and gender, marital status and income as independent variables. The male gender was a significant predictor for both anxiety, *p* < 0.001, OR = 2.83, 95%CI = (1.89, 4.24) and depression, *p* = 0.003, OR = 1.74, 95%CI = (1.21, 2.50). For the DS-14 scale, none of the above variables were predictive for D type personality. However, by applying multivariate linear regression according to the Enter method, using the DUKE scores as dependent variable, and age, gender, marital status and income as independent variables, age and marital status proved significant predictors for psychological stress. (Table 14).

## 4. Discussion

The present study was conducted on 500 CAD patients in stable condition divided into two groups: 200 patients who had received coronary artery bypass grafting (CABG) or percutaneous transluminal coronary angioplasty (PTCA) (Group 1) and 300 patients without myocardial revascularization (Group 2) with stable angina or thrombolysis. The patients in Group 1 underwent coronary artery bypass grafting (*n* = 119) or percutaneous transluminal coronary angioplasty (*n* = 81) before being admitted in the study. Besides usual determination of risk factors and functional investigations, we applied three different questionnaires of psychological stress in order to better individualize patients for cardiac rehabilitation. 

There were significant differences between the two groups regarding HAD-A (9.1 ± 4.18 for Group 1 vs. 7.8 ± 4.03 for Group 2, *p* = 0.002) and DUKE score (30.2 ± 12.25 for Group 1 vs. 22.7 ± 12.13 for Group 2, *p* < 0.001) after quantifying stress parameters by three different questionnaires. Our findings suggest that these symptoms are more prevalent in coronary patients who have undergone a revascularization procedure compared to no revascularization. 

No significant differences were found for HAD-D and type D personality (Table 1). Increased prevalence of both anxiety and depression symptoms in patients with CAD was found by Gu et al., before and after PTCA in a study conducted on 170 patients, aged 33–80 years old, who also sustained that the symptoms of anxiety and depression change over time in CAD patients, being more severe after PTCA [22]. 

In Group 1, there was a higher prevalence of male gender (78.2% in the CABG subgroup vs. 61.7% in the PTCA subgroup, *p* = 0.012). These results are in line with similar published findings [23,24]. 

In Group 1 with revascularization procedures, we observed significantly higher HAD-A scores in patients with PTCA vs. CABG (Chi2, *p* = 0.027). Numerous recent studies have demonstrated a relationship between CAD and anxiety [22,25], or both anxiety and depression, in patients who underwent myocardial revascularization interventions (CABG and PTCA), regardless of whether the questionnaires were applied before [25], after [26] or both before and after procedures [22]. We also observed that severe anxiety was significantly increased in patients with PTCA (Chi2 test, *p* = 0.027). Dahale et al. studied the association between CAD and anxiety and, based on the evidence, found that presence of anxiety is correlated with development and progression of coronary heart disease. The authors suggest that cardiologists should be trained to identify these patients for more effective management [27]. 

A significant number of risk factors contribute to the development and progression of CAD, including hypertension, dyslipidemia, smoking and diabetes [28]. By applying HADS in this study, we observed that for Group 1 (*n* = 200) increased values of HAD-A were significantly correlated, directly and weakly, with increased values of HbA1c (ρ = 0.23 and *p* = 0.001), and significantly, inversely and weakly with low eGFR values (ρ = −0.143 and *p* = 0.043). Increased HAD-D values were significantly correlated, directly and weakly, with high blood glucose values (ρ = −0.151 and *p* = 0.033) and indirectly with low values of eGFR (ρ = −0.16 and *p* = 0.024). There were no significant correlations between HAD-A and/or HAD-D and hypertension, dyslipidemia or smoking. The findings of this study showed that anxiety and/or depression were associated with the presence of comorbid conditions for CAD patients, like type 2 diabetes mellitus (*p* = 0.001 for HAD-A scores) or chronic kidney disease (*p* = 0.043 for HAD-A scores and *p* = 0.024 for HAD-D scores) (Table 11). The results are similar to the findings of Sandeep Chopra et al., in a study conducted on 60 CAD patients [29].

A comparison of risk factors between Group 1 (*n* = 200) and Group 2 (*n* = 300) showed significant differences for male gender (*p* < 0.001), smoking (*p* < 0.001), obesity (*p* = 0.001), total cholesterol (*p* < 0.001), HDL-c (*p* < 0.001), LDL-c (*p* < 0.001), triglycerides (*p* = 0.001) and family medical history of premature CVD (*p* = 0.043) (Table 1 and Table 2). Parkenson GR Jret al. classified the cardiovascular risk of adult primary care patients by self-reported quality of life based on their DUKE profile and found that the highest risk patients were more likely to be women and older [30]. In another study, LR Wu et al. observed that female gender, pain and disability are more predictive of depression and anxiety than any other medical illnesses, supporting that the DUKE screening instrument, which combines key symptoms of anxiety and depression, can be used in primary care screening [31]. Until now, no studies that used the DUKE scale for screening anxiety and depression in cardiovascular patients were reported in coronary artery disease, supporting the uniqueness of the current study.

After applying the DS-14 scale, we observed that only DS-14 NA scores were significantly increased in revascularized coronary patients with T2DM (*n* = 62) (Mann–Whitney test, *p* = 0.024). The association between the presence of T2DM and type D personality was significant (Chi2 test, *p* = 0.010). Kupper and Dennolet, in a review that synthesizes recent research findings, observed that in patients with CAD, type D personality is an established independent risk marker for worsening clinical and patient-reported outcomes [32]. Furthermore, Wang et al., examining patients treated with a drug-eluting stent, showed that patients with type D personality had a more than doubly increased risk of in-stent restenosis at 1 and 2 years after PTCA, independent of comorbidities [33].

Analyzing patients with severe anxiety in both groups (*n* = 12 in Group 1 vs. *n* = 32 in Group 2), we observed that those without myocardial revascularization and without income (*n* = 6) have a significantly increased HAD-A score compared to those with income (*n* = 26, *p* < 0.001) (Table 6). Moreover, we observed that in coronary patients with myocardial revascularization without a partner (*n* = 5), HAD-A (*p* = 0.041) and DUKE (*p* = 0.037) scores were significantly different than in those with a partner (*n* = 7). Significant differences were also observed in coronary patients without myocardial revascularization with a partner (*n* = 18) and without a partner (*n* = 14) in terms of HAD-A (*p* = 0.049) and DUKE (*p* = 0.042) scores (Table 7). In a study performed by Rodrigues et al. on preoperative patients submitted to their first cardiac surgery, after considering the age group, the differences found in terms of the presence of anxiety and depression symptoms were not statistically significant [34]. After we applied multinomial regression according to the Cox and Snell R-square model including all patients (*n* = 500) using gender, marital status and income as independent variables we found that for the HAD-A and HAD-D scores as dependent variables, male gender was a significant predictor for both anxiety, *p* < 0.001 and depression, *p* = 0.003. These results may be related to the fact that in Group 1 male gender was 71.55%, significantly prevalent (*p* < 0.001). The results of a multivariate adjusted model in a descriptive cross-sectional study, conducted by Rouhi Balasi et al. on 148 patients, showed that for the HAD scale education level was significantly associated with mild depression. Sex and age were significant predictors for severe depression. Male patients were less likely to have severe depression compared to females. Comparing age, they observed that middle-aged patients (45–64 years) were more likely to be diagnosed with severe depression than the elderly [35]. In our study for the DS-14 scale, none of the above variables were predictive for D type personality. However, by applying multivariate linear regression according to the Enter method, using the DUKE scores as dependent variable, and age, gender, marital status and income as independent variables, age and marital status proved significant predictors for psychological stress (Table 14).

Zhang P. studied the involvement of anxiety/depression after percutaneous coronary intervention on 150 CAD patients divided in two groups: stent group (*n* = 100) and non-stent group (*n* = 50 cases). Half of the cases in the stent group (*n* = 50) were treated with psychological methods (intervention group) and the other half untreated (non-intervention group). The findings showed no significant differences between biochemical criteria, anxiety scores or depression scores between groups before PTCA. After percutaneous coronary intervention, anxiety/depression scores in the stent group were significantly higher than in those from non-stent group (*p* < 0.05). There were no significant differences between the intervention group and the non-intervention group on anxiety/depression scores (*p* > 0.05). On the day of discharge, the anxiety/depression scores were the lowest in the intervention group (*p* < 0.05). The results of the study showed that PTCA can increase anxiety/depression, but appropriate psychological intervention can reduce the negative emotions [36].

Understanding anxiety and depression makes it possible to prevent cardiovascular disease. The high prevalence of depression and anxiety in CAD patients and the relationship with lifestyle change requires the integration of methods to identify and minimize adverse effects of depression and anxiety in cardiac rehabilitation and prevention programs [37,38]. For a better understanding of the importance of these parameters, a thorough history to clarify whether the occurrence of anxiety or depression in these patients was prior to onset of coronary artery disease may be beneficial, especially in those without previous anxiolytic and antidepressant treatment. In our study this type of medication was more significantly used in the group without myocardial revascularization (*p* = 0.039), but the high prevalence of anxiety in both groups is suggestive for the need of psychological counseling and/or medical treatment of these conditions, as well as long-term follow-up for best results during rehabilitation. Comprehensive programs also include the presence of a team of psychologists specialized in such techniques, as well as options for meditation and mindfulness based on stress reduction. These programs can also be associated with other predictive scores, like the Duke treadmill score for better patient follow-up [39].

## 5. Conclusions

The results obtained in this study emphasize that behavioral and psychological stress responses in patients with CAD do not only correlate with risk factors, but that other factors like gender, income and marital status also seem to play a decisive role, as shown by our multinomial and multivariate linear regression analyses. Each patient’s biological and health parameters including personality traits and socio-economic aspects may contribute to the course of cardiovascular disease. The three anxiety/depression questionnaires used, HADS, DUKE and the DS-14 scale, have proven useful to identify coronary patients in need of special psychological care and should be introduced as additional means of evaluation for a better individualization of rehabilitation programs. Furthermore, these results provide a framework for monitoring anxiety, depression and type D personality because comprehensive cardiac rehabilitation, with its global approach, offers the perfect setting to diagnose and help patients to cope with their emotional disorders. Psychological stress could have a relevant role in the outcomes of cardiac rehabilitation, which is related to progression of the disease. Spending time in rehabilitation groups gives patients the opportunity to understand the influence of psychological and biological risk factors, and to share responsibility in building strategies to manage daily stress. The questionnaires should also be applied at the end of the rehabilitation programs, for comparison, as proof of beneficial effects of these programs on all aspects of health.

## Figures and Tables

**Table 1:** Clinical, biochemical and demographic features of all coronary patients (*n* = 500).

Variable	Mean ± SD		Median (Min–Max)		Mean Rank		*p* ^sig^
	With Revasc. (*n* = 200)	Without Revasc. (*n* = 300)	With Revasc. (*n* = 200)	Without Revasc. (*n* = 300)	With Revasc. (*n* = 200)	Without Revasc. (*n* = 300)	
Age, years	62.3 ± 7.63	64.2 ± 7.92	66 (41–78)	64 (36–75)	235.23	273.40	0.004 ^s^
HAD-A	9.1 ± 4.18	7.8 ± 4.03	8 (0–18)	8 (1–21)	266.94	225.85	0.002 ^s^
HAD-D	7.4 ± 3.26	7.3 ± 3.47	8 (0–16)	8 (0–17)	251.83	248.50	0.799 ^ins^
SC DUKE	30.2 ± 12.25	22.7 ± 12.13	19 (2.7–50.7)	26.9 (2.7–58.2)	285.52	197.98	<0.001 ^s^
DS-14 SI	7.6 ± 7.28	7.6 ± 7.22	5 (0–28)	4 (0–28)	250.34	250.74	0.976 ^ins^
DS-14 NA	14.8 ± 8.99	13.4 ± 9.01	13 (0–28)	16 (0–28)	259.62	236.83	0.082 ^ins^
TC, mg/dL	167.5 ± 40.36	195.7 ± 49.49	164 (87–346)	190 (102–384)	199.48	284.51	<0.001 ^s^
HDL-c, mg/dL	43.5 ± 13.29	51.7 ± 13.17	42 (20–85)	52 (24–92)	194.59	287.78	<0.001 ^s^
LDL-c, mg/dL	93 ± 27.77	112.6 ± 37.51	90 (32–187)	105 (40–274)	203.93	281.55	<0.001 ^s^
TG, mg/dL	136 ± 71.08	158.3 ± 99.32	137 (53–890)	117.5 (38–491)	232.37	277.69	0.001 ^s^
Fast blood glucose, mg/dL	108.6 ± 34.99	110.4 ± 37.77	99.5 (62–322)	100 (60–314)	249.38	251.25	0.887 ^ins^
HbA1c, %	6.1 ± 0.84	6.1 ± 0.89	5.8 (5–10)	5.9 (5.1–10.5)	259.48	237.03	0.088 ^ins^
SBP, mmHg	136.3 ± 22.14	148.6 ± 23.63	130 (90–220)	145 (90–220)	204.15	281.40	<0.001 ^s^
DBP, mmHg	77.7 ± 11.97	86.1 ± 13.06	80 (50–120)	90 (55–128)	193.48	288.52	<0.001 ^s^
UA, mg/dL	5.3 ± 1.39	5.8 ± 1.23	6 (3.1–10.8)	5.05 (1.9–9.3)	226.14	287.04	<0.001 ^s^
Creatinine, mg/dL	1.1 ± 0.3	1.3 ± 0.26	1.2 (0.7–2.7)	1.07 (0.6–2.7)	217.06	300.66	<0.001 ^s^
eGFR, mL/min/1,73 m^2^	58.3 ± 13.99	55.8 ± 15.17	54 (25–100)	58 (18–110)	262.88	231.94	0.019 ^s^

^sig^—signification; ^s^—significant difference; ^ins^—insignificant difference; HAD-A, Hospital Anxiety and Depression Scale for Anxiety; HAD-D, Hospital Anxiety and Depression Scale for Depression; SC DUKE, Duke Anxiety-Depression Scale; DS-14 SI, Type D personality scale, social inhibition, DS-14 NA; Type D personality scale, negative affectivity; SBP, systolic blood pressure; DBP, diastolic blood pressure; eGFR, estimated glomerular filtration rate; UA, uric acid; HbA1c %, glycated haemoglobin; TC, total cholesterol; HDL-c, high-density lipoprotein cholesterol; LDL-c, low-density lipoprotein cholesterol; TG, triglyceride; T2DM, type 2 diabetes mellitus.

**Table 2:** Psychological and cardiovascular risk factors comparison between groups (Chi2 test).

Variable	*n* (%)		*p* ^sig^
	With Revasc. (*n* = 200)	Without Revasc. (*n* = 300)	
Male gender	143 (71.55)	114 (38.00)	<0.001 ^s^
Smoking (YES)	57 (28.50)	186 (62.00)	<0.001 ^s^
T2DM	62 (31.00)	107 (34.70)	0.394 ^ins^
Obesity (YES)	113 (56.50)	124 (41.30)	0.001 ^s^
Family medical history of premature CVD (YES)	88 (44.00)	105 (35.00)	0.043 ^s^
Previous anxiolytic and antidepressant treatment (YES)	22 (11.00)	52(17.33)	0.039 ^s^

^sig^—signification; ^s^—significant difference; ^ins^—insignificant difference.

**Table 3:** Comparison of CAD patients with coronary artery bypass grafting (CABG, *n* = 119) and percutaneous transluminal coronary angioplasty (PTCA, *n* = 81) regarding rank values of Hospital Anxiety and Depression Scale (HADS), Duke Anxiety-Depression Scale (DUKE) and Type D Personality Scale (DS-14) using the Mann–Whitney test.

Scale	CAD	Mean Rank	Sum of Ranks	*p* Value
HAD-A	CABG (*n* = 119)	91.91	10,937.50	0.010 ^s^
	PTCA (*n* = 81)	113.12	9162.50	
HAD-D	CABG (*n* = 119)	95.13	11,321.00	0.110
	PTCA (*n* = 81)	108.38	8779.00	
DUKE	CABG (*n* = 119)	94.60	11,257.00	0.048 ^s^
	PTCA (*n* = 81)	109.17	8843.00	
DS-14 SI	CABG (*n* = 119)	97.43	11,594.00	0.357
	PTCA (*n* = 81)	105.01	8506.00	
DS-14 NA	CABG (*n* = 119)	97.13	11,558.50	0.316
	PTCA (*n* = 81)	105.45	8541.50	

**Table 4:** Comparison of coronary patients from Group 1 with CABG (*n* = 119) and PTCA (*n* = 81) regarding severity of anxiety by applying HADS (Chi2 test).

CAD Group 1	HAD-A				Total
	Normal	Mild	Moderate	Severe	
CABG (*n* = 119)	48	54	14	3	119
	40.3%	45.4%	11.8%	2.5%	100.0%
PTCA (*n* = 81)	23	36	13	9	81
	28.4%	44.4%	16.0%	11.1%	100.0%
*p* value	0.115	0.996	0.521	0.027^s^	-

^s^—significant difference.

**Table 5:** Coronary patients from Group 2 regarding severity of anxiety by applying HADS (*n* = 300).

HAD-A	Absolute Frequency	Relative Frequency (%)	Cumulative Frequency
Normal	83	27.7	49.0
Mild	121	40.3	100.0
Moderate	64	21.3	21.3
Severe	32	10.7	59.7

**Table 6:** Current employment situation for patients with severe anxiety in both groups.

Characteristics	With Revasc. (*n* = 12)			Without Revasc. (*n* = 32)		
	With Income (*n* = 10)	Without Income (*n* = 2)	*p* Value	With Income (*n* = 26)	Without Income (*n* = 6)	*p* Value
Total pts	10 (85%)	2 (15%)	0.028 ^sa^	26 (81.8%)	6 (18.2%)	0.033 ^sa^
Female sex	7(87.5%)	1 (12.5%)	0.019 ^sa^	15 (83.3%)	3 (16.6%)	0.030 ^sa^
HAD-A	15.7 ± 1.16	16.8 ± 0.98	0.209 ^b^	15.5 ± 0.52	17.8 ± 1.68	<0.001 ^sb^
HAD-D	9.7 ± 1.16	9.6 ± 3.21	0.928 ^b^	10.2 ± 2.54	11.2 ± 2.59	0.383 ^b^
DUKE	22.2 ± 2.84	19.9 ± 11.68	0.482 ^b^	27.1 ± 12.79	23.3 ± 10.57	0.520 ^b^
DS-14 IS	9.3 ± 4.62	14.3 ± 9.01	0.373 ^b^	8.6 ± 8.46	12.8 ± 9.76	0.209 ^b^
DS-14 AN	23.0 ± 6.25	22.2 ± 6.98	0.864 ^b^	24.9 ± 3.71	25.9 ± 4.49	0.343 ^b^

^a^—Chi2 Test; ^b^—Mann-Whitney U Test; ^s^—signification.

**Table 7:** Marital status for patients with severe anxiety in both groups.

Characteristics	With Revasc. (*n* = 12)			Without Revasc. (*n* = 32)		
	Has a Partner (*n* = 7)	No Partner (*n* = 5)	*p* Value	Has a Partner (*n* = 18)	No Partner (*n* = 14)	*p* Value
Total pts	7 (60.0%)	5 (40.0%)	0.558 ^a^	18 (55.5%)	14 (44.5%)	0.705 ^a^
Female sex	5 (62.5%)	3 (37.5%)	0.545 ^a^	14 (58.3%)	10 (41.7%)	0.676 ^a^
HAD-A	15.8 ± 0.72	17.2 ± 0.75	0.041 ^sb^	15.1 ± 0.99	17.4 ± 1.98	0.049 ^sb^
HAD-D	9.2 ± 3.43	10.0 ± 2.19	0.699 ^b^	10.3 ± 2.34	11.1 ± 2.72	0.552 ^b^
DUKE	26.1 ± 10.19	14.9 ± 6.74	0.037 ^sb^	27.0 ± 11.92	15.5 ± 11.32	0.042 ^sb^
DS-14 IS	11.2 ± 9.60	15.0 ± 6.97	0.240 ^b^	10.3 ± 9.72	11.6 ± 9.35	0.687 ^b^
DS-14 AN	24.2 ± 4.40	20.7 ± 8.19	0.896 ^b^	25.0 ± 3.86	25.6 ± 4.38	0.513 ^b^

^a^—Chi2 Test; ^b^—Mann-Whitney U Test; ^s^—signification.

**Table 8:** Frequency table for coronary patients from Group 2regarding severity of depression by applying HADS (*n* = 300).

HAD-D	Absolute Frequency	Relative Frequency (%)	Cumulative Frequency
Normal	120	40.0	53.7
Mild	135	45.0	100.0
Moderate	41	13.7	13.7
Severe	4	1.3	55.0

**Table 9:** Risk analysis for severe depression according to marital status (*n* = 500) using chi square test.

		Severe Depression		Total
		Yes	No	
Marital status	No	5	83	88
		5.7%	94.3%	100.0%
	Yes	2	410	412
		0.5%	99.5%	100.0%
Total		7	493	500
		1.4%	98.6%	100.0%

**Table 10:** Risk analysis for severe depression according to low income (*n* = 500) using chi square test.

		Severe Depression		Total
		Yes	No	
Low income	No	6	34	40
		15.0%	85.0%	100.0%
	Yes	19	441	460
		4.1%	95.9%	100.0%
Total		7	25	475
		1.4%	5.0%	95.0%

**Table 11:** Correlations between measured parameters for group 1 (*n* = 200) regarding score values of HADS, DUKE and DS-14 (Spearman nonparametric correlation).

Scale	Corelation	HF (NYHA)	TC	HDL-c	LDL-c	TG	Fasting Blood Glucose	HbA1c	BMI
**HAD-A**	Spearman’s rho	0.023	−0.010	−0.024	0.009	0.006	−0.002	0.230	−0.107
	*p* Value	0.746	0.887	0.741	0.904	0.929	0.982	0.001	0.131
**HAD−D**	Spearman’s rho	0.022	−0.052	−0.099	−0.038	0.006	0.151	0.040	−0.134
	*p* Value	0.755	0.464	0.162	0.589	0.935	0.033	0.576	0.058
**DUKE**	Spearman’s rho	−0.143	0.145	0.250	0.044	−0.137	−0.063	−0.128	0.084
	*p* Value	0.044	0.040	<0.001	0.536	0.053	0.378	0.071	0.234
**DS−14 SI**	Spearman’s rho	0.049	−0.131	−0.086	−0.107	−0.001	−0.013	0.096	−0.108
	*p* Value	0.492	0.065	0.228	0.131	0.992	0.859	0.176	0.128
**DS−14 NA**	Spearman’s rho	0.042	−0.002	−0.048	0.046	−0.026	−0.045	0.186	−0.065
	*p* Value	0.559	0.972	0.498	0.521	0.711	0.531	0.008	0.357

**Table 12:** Correlation between measured parameters for group 1 (*n* = 200) regarding score values of HADS, DUKE and DS-14 (Spearman nonparametric correlation).

Scale	Corelation	HT Grades	SBP	DBP	LVEF	eGFR
HAD-A	Spearman’s rho	0.083	0.035	0.079	−0.086	−0.143
	*p* Value	0.241	0.619	0.269	0.225	0.043
HAD-D	Spearman’s rho	0.088	0.022	0.028	−0.065	−0.160
	*p* Value	0.217	0.753	0.697	0.359	0.024
DUKE	Spearman’s rho	−0.140	0.075	0.156	0.279	0.272
	*p* Value	0.048	0.294	0.027	<0.001	<0.001
DS-14 SI	Spearman’s rho	0.040	−0.029	0.051	−0.007	−0.103
	*p* Value	0.571	0.685	0.477	0.925	0.147
DS-14 NA	Spearman’s rho	0.043	−0.012	0.011	−0.062	−0.109
	*p* Value	0.541	0.866	0.881	0.380	0.124

**Table 13:** Correlation between presence of type 2 diabetes mellitus for group 1 (*n* = 200) and score values of HADS, DUKE and DS-14 (by Mann-Whitney Test).

Scale	T2DM	*n*	Mean Rank	Sum of Ranks	*p* Value *sig.*
HAD-A	NO	138	92.26	12,731.50	0.002 ^s^
	YES	62	118.85	7368.50	
HAD-D	NO	138	97.70	13,483.00	0.305 ^ins^
	YES	62	106.73	6617.00	
DUKE	NO	138	101.54	14,012.00	0.704 ^ins^
	YES	62	98.19	6088.00	
DS-14 SI	NO	138	96.17	13,272.00	0.111 ^ins^
	YES	62	110.13	6828.00	
DS-14 NA	NO	138	94.33	13,017.50	0.024 ^s^
	YES	62	114.23	7082.50	

^sig^—signification; ^s^—significant difference; ^ins^—insignificant difference.

**Table 14:** Multivariate linear regression (Enter method), *n* = 500. Coefficients ^a^.

Model	Unstandardized Coefficients		Standardized Coefficients	*t*	*Sig.*
	B	Std. Error	Beta		
Constant	36.706	5.538		6.628	0.000
age	−0.212	0.074	−0.130	−2.844	0.005
income	0.950	2.111	0.020	0.450	0.653
marital status	2.544	1.519	0.076	1.675	0.045
gender	0.587	1.131	0.023	0.519	0.604

a. Dependent variable: DUKE score.
